# Endogenous Plant Nitrous Oxide Formation: Evidence, Mechanisms and Ecosystem Implications

**DOI:** 10.3390/plants15162460

**Published:** 2026-08-13

**Authors:** Siddique Ahmad, Wenjing Song, Zhengyang Song, Shabnam Hadi, Mengdi Niu, Lingling Ma, Siying Wang, Laiba Urooj, Shuping Xiong, Zhiyong Zhang, Xiaochun Wang, Huiqiang Li, Xinming Ma, Yihao Wei

**Affiliations:** 1 College of Agronomy, Henan Agricultural University, Zhengzhou 450046, China; siddiqueb284@gmail.com (S.A.);; 2 Department of Biochemistry, Abdul Wali Khan University Mardan, Mardan 23200, Pakistan; 3 College of Life Sciences, Henan Agricultural University, Zhengzhou 450002, China

**Keywords:** nitrous oxide, plants, endogenous production, mechanisms, ecosystem

## Abstract

Nitrous oxide (N_2_O) is a potent greenhouse gas and a major ozone-depleting substance of the nitrogen cycle. While global N_2_O budgets focus on microbial soil sources, evidence suggests that living plants can also contribute to N_2_O emissions. However, the origin of plant-associated N_2_O fluxes is contested: measured emissions may arise from endogenous plant metabolism or merely from transport of soil-derived N_2_O. In this review, we clarify these pathways and synthesize current findings from laboratory and field studies. Experimental evidence from ^15^N-tracer and axenic-culture studies supports N_2_O formation linked to nitrate (NO_3_^−^) and nitrite (NO_2_^−^) in photosynthetic organisms, although mechanistic resolution varies among taxa. In angiosperms, proposed plastid/chloroplast and hypoxia-associated mitochondrial routes are linked to NO_3_^−^/NO_2_^−^ metabolism and NO formation, but the terminal NO-to-N_2_O step remains unresolved and differs from the flavodiiron-dependent mechanism demonstrated in algae. We discuss key environmental and physiological controls (substrate availability, light, O_2_ status, reductant supply) on these processes and highlight methodological challenges in source attribution (chamber artefacts, destructive sampling, isotope analysis). Our analysis suggests that plant-associated N_2_O fluxes are real, but highly variable and context-dependent. Rather than assuming generic “plant emission factors”, we emphasize source-specific approaches that distinguish endogenous formation from soil-derived transport and microbial production. This framework can improve N_2_O budgets and guide mitigation by indicating whether management should target soil N availability and microbial processes or physiological conditions favouring plant-associated N_2_O formation.

## 1. Introduction

Nitrous oxide (N_2_O) occupies a disproportionately important place in the Earth system. Although present in trace concentrations, it is a long-lived greenhouse gas and a major ozone-depleting substance, making it relevant to both climate forcing and stratospheric chemistry [1,2,3]. As a result, identifying the biological and physicochemical processes that control N_2_O production, reduction, transport, and storage remains a central task in biogeochemistry, agronomy, and atmospheric science. Most work has focused on soils, sediments, and aquatic systems because microbial nitrification, denitrification, nitrifier denitrification, and related pathways are widely recognized as dominant sources in terrestrial environments [4,5]. That emphasis has been productive, but it has also encouraged an overly soil-centred interpretation of plant involvement in N_2_O exchange.

Within this conventional framework, plants are usually treated as indirect modifiers of soil N_2_O dynamics or as conduits that transport externally produced N_2_O to the atmosphere. Plants can indeed alter N_2_O exchange by changing nitrate and ammonium availability, supplying carbon to the rhizosphere, modifying soil aeration and moisture, and moving gases through roots, stems, xylem, aerenchyma, and leaves [6,7,8]. However, that interpretation is not sufficient for every system. A growing body of work suggests that plant-associated tissues and photosynthetic eukaryotic cells may also generate N_2_O under specific physiological conditions, especially when nitrate or nitrite (NO_2_^−^) metabolism, oxygen (O_2_) availability, redox balance, or nitric oxide (NO) turnover becomes perturbed [9,10,11,12]. The central question is therefore no longer simply whether plants matter for N_2_O exchange, but how they matter mechanistically.

Several lines of evidence motivate that shift in framing. Early physiological and genetic studies showed that soybean (*Glycine max* L.) and winged bean (*Psophocarpus tetragonolobus* L.) released both NO and N_2_O during nitrate-reductase (NR) assays, that transgenic tobacco (*Nicotiana tabacum* L.) with impaired NO_2_^−^ reductase emitted more N_2_O than control plants, and that wheat (*Triticum aestivum* L.) leaves emitted N_2_O during nitrate assimilation in experiments supported by ^15^N labelling, chloroplast assays, and enzyme measurements [9,13,14]. Later work broadened the discussion beyond herbaceous crop tissues. Stem and leaf studies in woody species demonstrated that trees can be active components of ecosystem N_2_O exchange, particularly under flooding or changing physiological activity, even if those measurements alone cannot distinguish intracellular formation from transport of soil-derived N_2_O [15,16,17]. Axenic algal systems provide an additional and mechanistically valuable line of evidence because they minimize ambiguity arising from associated microorganisms. N_2_O production has been observed in *Chlorella vulgaris*, while studies in *Chlamydomonas reinhardtii* have directly linked NO reduction to N_2_O with photosynthetic electron transport and flavodiiron (FLV) proteins [18,19,20,21]. However, this mechanism is taxonomically restricted and should not be extrapolated directly to angiosperms, in which native FLV proteins are absent [22]. Algal studies therefore demonstrate the biochemical feasibility of endogenous N_2_O formation in photosynthetic eukaryotes but do not establish an equivalent terminal mechanism in flowering plants.

The difficulty is that plant-associated N_2_O exchange is intrinsically a source attribution problem [11,12]. In intact plant–soil systems, an observed flux may reflect endogenous formation within plant cells, production by epiphytic or endophytic microorganisms, conduit transport of N_2_O formed in soil or water, transient storage and delayed release within tissues, net uptake, or abiotic surface-associated reactions [10,23]. Recent in situ evidence further showed that foliar N_2_O can originate from endophytic bacteria, emphasizing the need to distinguish plant-cellular formation from plant-associated microbial production [24]. These processes can overlap in time and space, and a chamber flux alone cannot distinguish among them. This distinction matters scientifically because each pathway implies a different mechanism, a different set of controls, and a different meaning for ecosystem and inventory interpretation [12,25,26]. A review intended for a broader readership must therefore define these categories clearly, evaluate evidence against them explicitly, and avoid treating all plant-associated fluxes as mechanistically equivalent.

Current mechanistic evidence points most strongly to two non-exclusive intracellular routes, both of which require careful hedging [10]. One is a plastid or chloroplast-associated pathway linked to NO_3_^−^ assimilation, in which excess NO_2_^−^, NO, or related intermediates generated during imbalanced assimilatory metabolism enter side reactions that yield N_2_O [9]. The other is a hypoxia-linked mitochondrial pathway, in which NO_2_^−^ derived NO forms under low O_2_ conditions and may then be reduced further to N_2_O by respiratory components [11,27,28]. These candidate pathways are supported at the level of metabolic association in angiosperms, but their terminal N_2_O-forming reactions remain unresolved. Mechanisms demonstrated specifically in algae, particularly FLV-dependent NO reduction, should therefore be presented as comparative evidence rather than as established angiosperm pathways.

This review addresses plant-associated N_2_O as a problem of evidence ranking and source partitioning. It has four aims. First, it evaluates the evidence that plant cells or closely related photosynthetic eukaryotic cells can generate N_2_O endogenously. Secondly, it synthesizes the biochemical pathways most plausibly involved while distinguishing demonstrated steps from unresolved ones. Thirdly, it identifies the environmental and physiological conditions under which endogenous formation is most likely to become relevant. Fourthly, it outlines the experimental designs needed to separate endogenous formation from microbial production, conduit transport, storage, uptake, and abiotic surface reactions. The working hypotheses are that endogenous N_2_O formation is real but strongly context-dependent, that plastid/chloroplast and hypoxia-associated mitochondrial processes are the leading mechanistic candidates, and that the main barrier to progress is now rigorous source attribution rather than lack of biological plausibility. Clarifying these points should sharpen both plant physiological understanding and the treatment of plant-associated N_2_O in ecosystem-scale assessments.

## 2. Endogenous Formation Versus Conduit Transport

The term plant-derived N_2_O requires precise use because observed fluxes can arise from distinct processes with different meanings. Here, plant-associated N_2_O exchange is partitioned into endogenous formation, plant-associated microbial production, and conduit transport, while also recognizing storage and release behaviour and surface-associated reactions as interpretive confounders. These processes can co-occur in intact plant–soil systems, but they differ in source, mechanism, and relevance to plant physiology and ecosystem budgets. Endogenous formation refers to N_2_O produced by chemical or enzymatic reactions inside plant cells, or in related photosynthetic eukaryotic cells [9,11,18]. Support is strongest when N_2_O production tracks NO_3_^−^, NO_2_^−^, NO, or respiratory electron transport. Evidence from transgenic tobacco (*N. tabacum*), wheat (*T. aestivum*) leaves, axenic *Chlorella*, and *Chlamydomonas* indicates that photosynthetic cells can generate N_2_O under defined conditions [14,19]. Plant-associated microbial production refers to N_2_O generated by epiphytic or endophytic microorganisms on or within plant tissues. In that case, the plant supplies carbon, nitrogen (N) compounds, habitat, or O_2_ gradients, but the immediate N_2_O-forming step is microbial rather than plant cellular metabolism [4,5,10,12]. Conduit transport refers to N_2_O produced externally in soil or water, then released through roots, stems, leaves, or shoots. Rice (*Oryza sativa* L.) and wetland plants can move gases through aerenchyma, while trees and non-aerenchymatous crops may also transport N_2_O by diffusion, xylem flow, or internal gas spaces [6,7,15,16,29]. Consequently, chamber-scale plant fluxes should be treated as composite signals until source attribution separates endogenous formation from microbial production and transport; storage, delayed release, and surface reactions may contribute.

## 3. Endogenous Plant-Associated N_2_O Formation

Evidence for endogenous plant-associated N_2_O formation is not equally strong across systems, and the section is clearer when organized by inference strength rather than by organism name alone. The most persuasive evidence comes from controlled angiosperm studies that directly connect N_2_O release with NO_3_^−^/NO_2_^−^ metabolism. NR assays in soybean (*G. max*) and winged bean (*P. tetragonolobus*) detected both NO and N_2_O, while antisense suppression of NO_2_^−^ reductase in tobacco (*N. tabacum*) increased N_2_O emission, indicating that disrupted NO_2_^−^ metabolism can increase reactive N intermediates associated with N_2_O formation [13,14]. Wheat (*T. aestivum*) studies strengthened this interpretation by showing greater N_2_O emission under NO_3_^−^ nutrition than under NH_4_^+^ nutrition, supported by ^15^N labelling and chloroplast-based evidence for a leaf N assimilation source [9]. These results justify mechanistic follow-up on a mitochondrial reductive pathway, but they do not by themselves prove every downstream step of that route.

A second strong line of evidence comes from axenic photosynthetic systems, which minimize microbial ambiguity. Axenic *Chlorella vulgaris* and *Chlamydomonas reinhardtii* produce N_2_O through pathways involving NO_2_^−^, NO, NR, and photosynthetic or respiratory electron flow [18,19,20,21]. Although algae are not vascular plants, they demonstrate intrinsic N_2_O-forming capacity in photosynthetic eukaryotic cells. In *Chlamydomonas*, the dual NR NO-forming NiR (NOFNiR) system catalyzes NO_2_^−^ reduction to NO, thereby supplying a possible substrate for downstream N_2_O-forming reactions [30]. Nevertheless, these detailed algal mechanisms should not be transferred directly to angiosperms. Although angiosperms contain amidoxime-reducing component (ARC)-related proteins that have been considered potential NOFNiR candidates, their physiological contribution to NO_2_^−^ dependent NO formation and their connection with N_2_O production remain unconfirmed.

By contrast, woody organ measurements provide ecologically important but mechanistically weaker evidence. Leaves, stems, and shoots can exchange N_2_O, especially under flooding, seasonal physiological activity, and developmental change [15,16,29]. Yet these fluxes may integrate endogenous production, soil-derived transport, microbial activity, storage, and consumption, so they should be interpreted as plant-mediated exchange unless cellular origin is independently verified.

^15^N tracing and isotopocule approaches are therefore most powerful when used as source-partitioning tools that bridge controlled physiology and intact systems [9,10,12]. These methods are especially valuable in field settings, but signatures can overlap among pathways and must be interpreted alongside microbial controls, physiological measurements, and transport assessments. The comparative strength of evidence across study systems, together with the principal interpretive limitations of each system, is summarized in Table 1. Taken together, the evidence supports endogenous N_2_O formation most strongly in controlled NO_3_^−^/NO_2_^−^ fed systems and less strongly in intact field systems, where source partitioning remains essential.

## 4. Plausible Mechanistic Pathways of Endogenous Plant-Associated N_2_O Formation

In angiosperms, NO_3_^−^ is first reduced to NO_2_^−^ mainly by cytosolic NR [36], using NADH or NADPH as electron donors. NO_2_^−^ then enters plastids or chloroplasts, where nitrite reductase (NiR) normally reduces it to NH_4_^+^ for amino acid synthesis [19]. NO can be produced in chloroplasts and mitochondria through oxidative and reductive reactions, so mechanistic discussion must separate demonstrated steps from inference [37,38]. At present, two intracellular routes are most plausible, whereas non-cellular surface reactions should be treated separately.

## 5. Chloroplast-Associated NO_3_^−^ and NO_2_^−^ Assimilation Route

**Figure 1:**
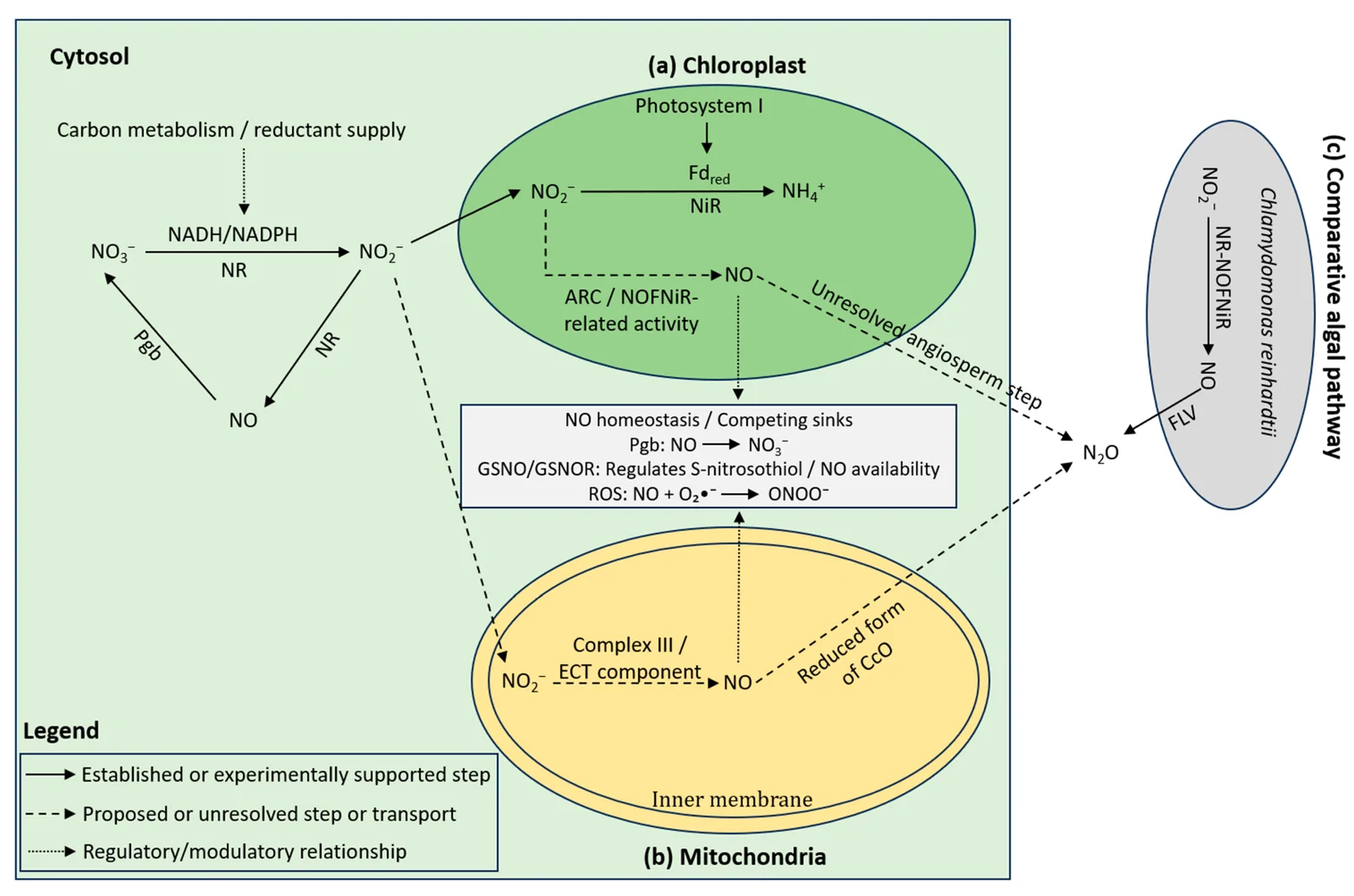
Proposed pathways of endogenous N_2_O formation in plants. (**a**) Candidate chloroplast pathway in angiosperms. (**b**) Candidate mitochondrial pathway in angiosperms. (**c**) Experimentally demonstrated pathway in *Chlamydomonas reinhardtii*. Abbreviations: ARC, amidoxime-reducing component; CcO, cytochrome c oxidase; ETC, electron transport chain; FLV, flavodiiron protein; Fd_red_, reduced ferredoxin; GSNO, S-nitrosoglutathione; GSNOR, S-nitrosoglutathione reductase; NH_4_^+^, ammonium; NiR, nitrite reductase; NO, nitric oxide; NO_2_^−^, nitrite; NO_3_^−^, nitrate; NOFNiR, nitric oxide-forming nitrite reductase; N_2_O, nitrous oxide; NR, nitrate reductase; O_2_•^−^, superoxide radical; ONOO^−^, peroxynitrite; Pgb, phytoglobin; ROS, reactive oxygen species.

The clearest first candidate is an assimilation-associated plastid or chloroplast route, in which NO_3_^−^ supplies NO_2_^−^ and NO to downstream N_2_O-forming chemistry (Figure 1a). Under oxygenated conditions, oxidative NO formation may proceed through L-arginine-, polyamine-, or hydroxylamine-dependent reactions, whereas NO_2_^−^ dependent reductive NO formation becomes more important when NO_2_^−^ accumulates or redox balance is disturbed [39,40]. In leaves, NiR uses photosystem I-derived reduced ferredoxin to reduce NO_2_^−^, linking nitrate assimilation with photosynthetic electron transport and carbon-nitrogen balance [41,42]. If NR supplies NO_2_^−^ faster than chloroplast NiR can consume it, especially under high nitrate supply or impaired NiR activity, excess NO_2_^−^ may enter side reactions that generate NO and possibly N_2_O [14,43]. Isotopic experiments recovered ^15^N-N_2_O from ^15^N-NO_3_^−^ or ^15^N-NO_2_^−^, but not from NH_4_^+^, linking emitted N_2_O to nitrate or NO_2_^−^ metabolism rather than to ammonium assimilation alone [9,13,31]. In wheat, nitrate assimilation increased N_2_O production, and intact chloroplasts and NiR preparations produced N_2_O, whereas NR alone did not, placing the likely N_2_O-forming step within chloroplast NO_2_^−^ metabolism rather than at cytosolic NR [9]. NiR-deficient tobacco also emitted N_2_O after NO_3_^−^ or NO_2_^−^ supply, while NR inhibition suppressed NO_3_^−^ derived N_2_O, indicating that NR mainly delivers substrate and that excess NO_2_^−^ favours N_2_O formation downstream [14,26,31]. NR can reduce NO_2_^−^ to NO [44], NO increases during NO_2_^−^ accumulation, NR-deficient *Chlamydomonas* shows impaired NO_2_^−^ dependent NO production, and soybean (*G. max*) chloroplasts contain NO [45]. NR-dependent NO metabolism may also be regulated by signalling pathways [46]. In Chlamydomonas reinhardtii, RAF14 and RAF79 are repressed by ammonium but induced by NO_3_^−^, NO_2_^−^ and N deprivation, suggesting roles in N-status signalling. A hypothetical model proposes that MAPK-dependent phosphorylation of NR may influence its interactions with NOFNiR or THB1 and thereby alter NO production or removal; however, this model remains unverified and its relevance to angiosperm N_2_O formation is unknown [47]. During NiR-mediated reduction in NO_2_^−^ to NH_4_^+^, NO or hydroxylamine-like intermediates may leak when electron flow and substrate supply are imbalanced [48].

An additional candidate linking NO_3_^−^/NO_2_^−^ metabolism with N_2_O formation is NOFNiR, formerly described as the ARC. In *Chlamydomonas reinhardtii*, NOFNiR receives electrons from the diaphorase and haem domains of nitrate reductase and catalyzes the reduction in NO_2_^−^ to NO. This NR-NOFNiR system therefore provides a mechanistically supported route by which NO_3_^−^/NO_2_^−^ metabolism can supply NO for subsequent N_2_O formation. Importantly, NOFNiR catalyzes the reduction in NO_2_^−^ to NO and should not be considered the enzyme responsible for the terminal NO-to-N_2_O reaction [30,49]. The terminal NO-to-N_2_O step in angiosperm chloroplast-associated metabolism remains unresolved and should not be presented as demonstrated enzymology; it may involve non-enzymatic chemistry or an unidentified NO-reducing activity [11]. In *Chlamydomonas reinhardtii*, by contrast, photosynthetic electron transport and chloroplast FLV proteins experimentally mediate the reduction in NO to N_2_O (Figure 1c) [20,21]. Because native FLV proteins are absent from angiosperms, this algal terminal mechanism cannot be assigned to flowering plants. Accordingly, the proposed angiosperm pathway contains two unresolved components: the physiological contribution of NOFNiR or alternative systems to NO generation and the identity of the terminal catalyst converting NO-to-N_2_O.

## 6. Hypoxia-Associated Mitochondrial Route

The second candidate is a hypoxia-associated mitochondrial route operating when O_2_ supply becomes limiting (Figure 1b). Under these conditions, the reductive pathway of NO formation becomes more important than the oxidative pathway. The mechanism by which NO_2_^−^ reaches mitochondria remains uncertain, but after cytosolic reduction in NO_3_^−^ to NO_2_^−^, transport through mitochondrial inner-membrane anion channels has been proposed as a plausible entry route [37]. Mitochondria from fungi, algae, and plants can reduce NO_2_^−^ to NO under hypoxic or anoxic conditions, showing that NO_2_^−^ can serve as an alternative electron acceptor in low O_2_ metabolism [49,50,51]. However, the specific enzymes contributing to mitochondrial NO production are not fully defined. Evidence instead supports participation of the electron transport chain under hypoxia [52], and in pea (*P. sativum*) mitochondria NO_2_^−^ reduction to NO has been associated with complex III [53]. Multiple components may contribute depending on redox state, O_2_ availability, and tissue physiology. Cytochrome c oxidase (CcO) is especially relevant because the reduced form of this enzyme can catalyze the reduction of NO to N_2_O [54], and isotopic work in higher organisms shows that CcO can indeed reduce NO to N_2_O [55]. The available evidence therefore supports the hypothesis that hypoxic mitochondrial metabolism may convert NO_2_^−^ to NO and then, under suitably reduced conditions, further reduce NO to N_2_O. Its quantitative importance in intact plants, and the extent to which it competes with NO scavenging and other sinks, remain unresolved. For that reason, studies invoking this pathway should measure N_2_O together with NO, NO_2_^−^, O_2_ status, respiration, mitochondrial redox state, cytochrome oxidase activity, and phytoglobin (Pgb) expression. Reported chloroplast and mitochondrial biological systems used to investigate NO reduction and associated N_2_O formation are compared in Table 2.

## 7. Abiotic and Surface-Associated N_2_O Formation

Abiotic or surface-associated N_2_O formation should be treated as a confounder, not an intracellular pathway. This distinction is essential because otherwise source-attribution claims can become easily conflated in intact-plant systems. UV-induced N_2_O emission from vegetation may arise from photochemical reactions involving nitrate, NO_2_^−^, or organic N compounds on leaf surfaces [57]. Such reactions may be favoured by strong radiation, dry surfaces, atmospheric N deposition, fertilizer residues, senescent tissues, or nitrate-containing particles. These processes can contribute to measured plant surface N_2_O fluxes, but they do not demonstrate endogenous production. Current evidence therefore supports a chloroplast-associated assimilation route and a conditional hypoxia-associated mitochondrial route, whereas surface reactions must be separated before plant cell N_2_O formation can be quantified confidently.

## 8. Physiological and Environmental Controls

Plant-associated N_2_O exchange is regulated by substrate supply, redox state, O_2_ availability, carbon metabolism, anatomy, and physiology. NO_3_^−^ fed wheat (*T. aestivum*) leaves emit more N_2_O than NH_4_^+^ fed leaves, and tobacco (*N. tabacum*) and algal studies show that disturbed NO_2_^−^ metabolism elevates N_2_O formation [9,14,18]. NR activity, NiR capacity and transient NO2^−^ accumulation are primary controls, but NR-dependent NO metabolism may also respond to regulatory signalling, including the proposed RAF14/RAF79-associated MAPK pathway in *Chlamydomonas* [47]. Light and carbon status modify this baseline through reductant supply, gas exchange, and respiration. Photosynthesis supports NO_3_^−^ assimilation [58], but high light does not increase N_2_O release automatically because illumination changes stomatal behaviour and plant–soil coupling [59]. Darkness or low light may favour respiration-associated routes by maintaining carbohydrate-derived electron supply and shifting organellar redox balance [60,61]. O_2_ availability is a second major filter. Flooding, waterlogging, compaction, senescence, dense stems, and high respiration can create hypoxic microsites [62] in which NO_2_^−^ becomes an alternative electron acceptor and is reduced to NO by mitochondrial components [11,23,37]. Endogenous N_2_O formation is therefore most plausible where NO_2_^−^ supply and reductive pressure coincide. A key regulatory node is competition between putative N_2_O formation and multiple pathways that consume or buffer NO. Under oxygenated conditions, oxygenated ferrous Pgb oxidizes NO to NO_3_^−^ as shown in Figure 1. Under prolonged hypoxia, limited O_2_ may weaken Pgb-mediated NO oxidation while NO_2_^−^ derived NO increases; if Pgb buffering is insufficient, NO may accumulate and favour N_2_O formation [11,37,63,64]. Pgb scavenges NO in the cytosol [28]. NO availability is also regulated through S-nitrosoglutathione (GSNO) formation and GSNOR-mediated turnover of cellular S-nitrosothiols; thus, GSNOR acts indirectly rather than as a direct NO scavenger [65]. NO can also react non-enzymatically with superoxide (O_2_•^−^) to form peroxynitrite (ONOO^−^), while antioxidant systems, including superoxide dismutase and peroxiredoxins, regulate ROS and reactive nitrogen species [38,66,67]. These pathways may affect the NO available for endogenous N_2_O formation, although their direct influence remains untested. Variation in Pgb abundance, S-nitrosothiol turnover, antioxidant capacity and tissue redox conditions may therefore contribute to plant-associated N_2_O exchange. Plants may also lower net ecosystem N_2_O by reducing soil NO_3_^−^, increasing N immobilization, or favouring complete denitrification to N_2_ [8].

## 9. Methodological Challenges in Source Attribution

The main problem is source attribution. Chamber measurements quantify net exchange, but they cannot determine whether observed N_2_O originated from plant cells, microorganisms, soil transport, tissue storage, consumption, or disturbance release [12,15,34,68,69]. Chamber data are screens, not mechanistic proof. Plant exchange may be bidirectional. Negative fluxes can reflect tissue entry, storage exceeding release, or consumption exceeding production. Such uptake should not be interpreted as biochemical assimilation without additional evidence, because gradients, stomata, transport, and metabolism contribute [10,17,70]. Destructive partitioning adds uncertainty. Cutting shoots, removing plants, or incubating excised tissues can alter transpiration, gradients, wounding, O_2_ status, microbial activity, and release of stored gases [71,72]. These approaches estimate exchange, but they cannot prove endogenous production without controls and source markers [12,15].

Stable-isotope source attribution can be strengthened by combining 15N labelling with N_2_O isotopocule analysis. Recovery of ^15^N-enriched N_2_O after ^15^NO_3_^−^ or ^15^NO_2_^−^ supply can link emissions to plant N metabolism when microbial sources are excluded or quantified [9,34]. Site preference (SP = δ^15^Nα − δ^15^Nβ) compares ^15^N enrichment at the central and terminal N atoms of N_2_O [35]. Pure-culture studies report SP values near 0‰ for bacterial denitrification [32] and approximately 37‰ for fungal denitrification [33], although fungal values may overlap with nitrification. Combining SP with δ^18^O in dual-isotopocule plots therefore improves source interpretation. Plant-emitted N_2_O has shown a fingerprint distinct from known microbial sources [10], while paired field chambers detected significantly different SP values for plant- and soil-emitted N_2_O [12]. Because signatures can overlap and N_2_O reduction can modify SP and δ^18^O, isotopocule evidence should be interpreted with microbial and transport controls [34,35]. Definitive validation of endogenous N2O pathways in angiosperms will require organelle-level enzyme assays, genetic loss-of-function/complementation studies, and intact-plant source attribution. Organelle-enriched fractions should be tested with ^15^N-labelled substrates while monitoring N_2_O, NO, O_2_ status and redox conditions, and candidate genes should be assessed using knockout and complemented lines [9,14]. Field studies should combine separate plant and soil chambers with ^15^N site-preference/isotopocule analysis and parallel microbial and transport controls [12]. Aseptic and axenic systems are essential for demonstrating potential, but lack field realism. They establish N_2_O formation in Chlorella and Chlamydomonas, but they do not define plant–soil flux magnitude [18,19]. Because plant fluxes are small and sensitive to enclosure time, light, temperature, humidity, pressure, leakage, and calibration, studies should report detection limits, blanks, leak tests, microbial controls, tissue condition, substrate concentrations, and normalization basis [9,68,69,73,74,75]. They should also explicitly state whether plant material remained intact and how microbial and physiological controls were applied [11,15,76].

## 10. Conclusions

Plant-associated N_2_O exchange should no longer be framed merely as a passive extension of soil microbial emissions. Available evidence supports a more precise view: plants can transport soil-derived N_2_O, while plant or plant-like photosynthetic cells can also form N_2_O under specific physiological conditions. The strongest evidence in angiosperms comes from controlled NO_3_^−^ fed wheat (*T. aestivum*) leaves and transgenic tobacco (*N. tabacum*) with impaired NO_2_^−^ reduction. However, algal mechanisms should not be directly extrapolated to flowering plants. In *Chlamydomonas reinhardtii*, the NR–NOFNiR system produces NO from NO_2_^−^, and FLV proteins subsequently reduce NO to N_2_O. In angiosperms, the role of ARC/NOFNiR-related proteins remains unconfirmed, FLV proteins are absent, and the terminal NO-to-N_2_O step remains unresolved. Therefore, chloroplast-associated NO_3_^−^/NO_2_^−^ metabolism and the hypoxia-linked mitochondrial route should be considered candidate pathways rather than fully demonstrated mechanisms. Their importance likely depends on N supply, O_2_ availability, carbon metabolism, tissue characteristics, and the balance among Pgb-mediated NO oxidation, GSNO/GSNOR-regulated S-nitrosothiol turnover, and ROS–antioxidant interactions. Their quantitative contribution remains uncertain because direct subcellular verification and field-scale source attribution are limited. Priority should therefore be given to organelle-level assays, genetic knockout/complementation studies, and intact-plant ^15^N isotopocule measurements with microbial and transport controls, particularly under high NO_3_^−^ availability, flooding, hypoxia, or strong plant-mediated gas transport. These priorities are especially relevant in crop, wetland, and woody systems where multiple source pathways may overlap. Taken together, current evidence supports endogenous plant N_2_O formation as a condition-dependent process with quantitative importance that remains unresolved.

## Figures and Tables

**Table 1:** Evidence for endogenous plant-associated N_2_O formation: observations, limitations, and validation approaches.

Study System	Key Observation	Main Limitation	Feasible Approach	References
Soybean (*G. max*) and winged bean (*P. tetragonolobus*) NR assays	NR assays produced both NO and N_2_O during NO_3_^−^ reduction.	Terminal N_2_O-forming reaction unresolved; assay side reactions possible.	Use ^15^N-NO_3_^−^/NO_2_^−^ with subcellular assays, simultaneous NO/N_2_O measurement, and controls.	[13]
NiR-impaired tobacco (*N. tabacum*)	NiR suppression increased N_2_O after NO_3_^−^/NO_2_^−^ supply; NR inhibition reduced NO_3_^−^-derived N_2_O.	Links N_2_O to disturbed nitrite metabolism but not the terminal reaction.	Combine genetic loss-of-function/complementation with organelle-level ^15^N and NO/N_2_O assays.	[14,31]
Wheat (*T. aestivum*) leaves/chloroplasts	More N_2_O under NO_3_^−^ than NH_4_^+^; ^15^N tracing and chloroplast/NiR assays supported N_2_O formation.	Terminal N_2_O-forming catalyst unidentified.	Couple ^15^N tracing with chloroplast/NiR assays and genetic testing of candidate reactions.	[9]
Axenic *C. vulgaris*	Axenic cultures produced N_2_O, demonstrating intrinsic photosynthetic N_2_O formation.	Algal evidence does not establish an angiosperm mechanism.	Screen mechanisms in algae, then test candidates in aseptic angiosperm cells or organelles.	[18]
Axenic *C. reinhardtii*	NO_2_^−^/NO metabolism supports N_2_O formation; NOFNiR supplies NO and FLVs reduce NO to N_2_O.	FLV-mediated reduction is algal and cannot be extrapolated to angiosperms.	Test ARC/NOFNiR-related and alternative NO-reducing activities in angiosperms.	[19,20,21,22,30]
Woody organs in intact systems	Stems, leaves, and shoots exchange N_2_O under flooding and physiological change.	Flux may combine endogenous, microbial, transport, storage, and uptake processes.	Use paired plant–soil chambers with ^15^N/isotopocule, microbial, and transport controls.	[15,16,17,29]
Paired plant–soil field chambers	Plant and soil N_2_O showed different site-preference signatures.	SP may overlap among pathways and change during N_2_O reduction.	Combine SP with δ^18^O/δ^15^N, ^15^N tracing, N_2_O/N_2_, and microbial/transport controls.	[12,32,33,34,35]

Abbreviations: ARC, amidoxime-reducing component; FLV, flavodiiron protein; NiR, nitrite reductase; NO, nitric oxide; NOFNiR, NO-forming nitrite reductase; NR, nitrate reductase; SP, site preference.

**Table 2:** Experimental evidence relevant to NO formation and NO-to-N_2_O reduction in chloroplast- and mitochondrial-associated pathways.

Biological Material/ System	Experimentally Supported Observation	Mechanistic Relevance/Limitation	References
Purified CcO systems (non-plant)	Reduced CcO converted NO to N_2_O under low-O_2_/anaerobic conditions.	Direct NO-reductase activity, but non-plant evidence does not establish a plant mitochondrial pathway.	[54,55]
Soybean (*G. max*) and winged bean (*P. tetragonolobus*) NR assays	NR assays detected NO and N_2_O during NO_3_^−^ reduction.	Terminal catalyst unresolved; assay side reactions remain possible.	[13]
NiR-impaired tobacco (*N. tabacum*)	NiR suppression increased N_2_O after NO_3_^−^/NO_2_^−^ supply.	Supports linkage to disturbed nitrite metabolism; terminal step unresolved.	[14]
Wheat (*T. aestivum*) leaves/chloroplasts/NiR	^15^N tracing linked N_2_O to nitrate assimilation; chloroplasts and NiR preparations produced N_2_O.	Strong chloroplast-level evidence; terminal catalyst remains unknown.	[9]
Aseptically cultured tobacco/wheat	^15^N-labelled N treatments yielded labelled N_2_O in aseptic plants.	Reduces microbial ambiguity, but the biochemical sequence remains unresolved.	[31]
Pea mitochondria under low O_2_	Mitochondria reduced NO_2_^−^ to NO; complex III was implicated.	Supports mitochondrial NO formation, not subsequent NO-to-N_2_O reduction.	[53]
*C. reinhardtii* NR–NOFNiR	NOFNiR reduced NO_2_^−^ to NO using NR-derived electrons.	Establishes upstream NO formation; NOFNiR is not the terminal N_2_O enzyme.	[30,49]
*A. thaliana* ARC1/ARC2	ARC2 reduced NO_2_^−^ to NO in vitro; its physiological role was not confirmed.	Shows angiosperm NO-forming potential, not endogenous N_2_O formation.	[56]
*C. reinhardtii* electron-flow/FLV system	N_2_O depended on NO_2_^−^/NO and electron flow; chloroplast FLVs mediated NO-to-N_2_O reduction.	Direct algal terminal mechanism; native FLVs are absent from angiosperms.	[19,20,21,22]

Abbreviations: ARC, amidoxime-reducing component; CcO, cytochrome c oxidase; FLV, flavodiiron protein; NiR, nitrite reductase; NOFNiR, NO-forming nitrite reductase; NR, nitrate reductase.

## Data Availability

No new data were created or analyzed in this study. Data sharing is not applicable to this article.
